# The effect of objective income and perceived economic resources on self-rated health

**DOI:** 10.1186/s12939-020-01304-2

**Published:** 2020-11-04

**Authors:** Catia Cialani, Reza Mortazavi

**Affiliations:** Economics Unit, School of Technology and Business Studies, Dalarna University, 791 88 Falun, Sweden

**Keywords:** Self-rated health status, Objective income, Perceived economic resources, Ordered probit model

## Abstract

**Background:**

Several studies have demonstrated that self-rated health status is affected by socioeconomic variables. However, there is little knowledge about whether perceived economic resources affect people’s health. The purpose of this study is to examine the relationship between self-rated health status and different measures of income. Specifically, the effect of both objective income and perceived economic resources are estimated for a very large sample of households in Italy. By estimating this relationship, this paper aims at filling the previously mentioned gap.

**Methods:**

The data used are from the 2015 European Health Interview Survey and were collected using information from approximately 16,000 households in 562 Italian municipalities. Ordinary and generalized ordered probit models were used in estimating the effects of a set of covariates, among others measures of income, on the self-rated health status.

**Results:**

The results suggest that the subjective income, measured by the perceived economic resources, affects the probability of reporting a higher self-rate health status more than objective income. The results also indicate that other variables, such as age, educational level, presence/absence of chronic disease, and employment status, affect self-rated health more significantly than objective income. It is also found that males report more frequently higher rating than females.

**Conclusions:**

Our analysis demonstrates that perceived income affects significantly self-rated health. While self-perceived economic resources have been used to assess economic well-being and satisfaction, they can also be used to assess stress levels and related health outcomes. Our findings suggest that low subjective income adversely affects subjective health. Therefore, it is important to distinguish between effects of income and individuals’ perceptions of their economic resources or overall financial situation on their health. From a gender perspective, our results show that females are less likely to have high rating than males. However, as females perceive an improved economic situation, on the margin, the likelihood of a higher self-rated health increases compared to males.

## Highlights

In the Italian context, it is found that perceived income affects self-rated health while objective income has no significant effect.

An improved economic situation increases the likelihood of a higher self-rated health more for females than males.

Respondents suffering from chronic disease report lower self-rated health than others.

Those with university education report higher self-rated health than others.

## Introduction

Self-rated health (SRH, also known as self-assessed health or self-perceived health) is a conventional measure of health status based on individuals’ expressed perceptions of their current personal health status. The perceptions are generally elicited using a survey question that asks respondents to rate their overall health on a four- or five-point scale ranging from poor to excellent. A typical question is “In general, would you say your health is excellent, very good, good, fair, or poor?” [8]. According to various researchers, socioeconomic status (SES), morbidity-related, lifestyle and psychosocial factors are the main determinants of SRH [39, 40, 46]. SES is usually measured in terms of income, education and employment levels [3, 21]. However, measures the objective variable, such as income, may be irrelevant if income does not reflect people’s perceptions of their financial status, as low perceived economic status can impair health, either directly through stress, or indirectly through adverse health-related behaviours [1]. Moreover, individuals’ ratings of their economic situations may depend on the social context, situations of others, or the own past living standards. Several studies have also shown that individuals differ in psychological responses to objective financial situations, and that the subjective dimension has stronger associations with health conditions [9, 26, 45]. Effects of socioeconomic status on health may also depend on individuals’ perceptions of their position in the social hierarchy [36]. Thus, the psychosocial impact of belonging to a particular social class influences individuals’ health, as well as absolute income level [51].

Despite the summarized advances in understanding, we know little about the relationship between health status and self-perceived income sufficiency (subjective income), which differs from objective income and is linked to fundamental behavioural economics issues. Subjective income, as measured by self-perceived income sufficiency or economic resources, is conceptualized as individuals’ personal assessment of their economic well-being [24]. It indicates their evaluations of the relationship between their objective income and expenses, more specifically its adequacy to meet personal or household goals. Questions regarding objective income focus on a specific income level, but do not cover household arrangements, debts, assets, and other relevant factors. In contrast, responses to questions on subjective income generally reflect individuals’ ability to meet their needs. From a psychological standpoint, self-perceived income sufficiency, as a measure of economic well-being focused on an individual’s life evaluation, is a component of an overall subjective assessment of an individual’s well-being or quality of life [13, 20]. From an economic standpoint, subjective measures capture the economic utility level reflecting an individual’s overall well-being or satisfaction, derived through maximization of her or his consumption of goods, services, and leisure within budgetary constraints.

A few studies have addressed the relationship between perceived financial hardship and SRH [5, 14, 42, 43], mainly for elderly people in a few countries such as India, Costa Rica, Hong Kong, China and Taiwan. However, apart from these studies, the link between perceived economic resources, or subjective financial well-being, and SRH has received little attention in the literature. The objective of the work presented here is to address this gap, by examining relationships between SRH status and traditional measures of socioeconomic variables, presence of chronic disease, and the influence of perceived economic resources in Italy. Our hypothesis is that people who perceive a lack of economic resources to meet their basic needs are more likely to have poor perceived SRH. Data were collected from the national Health Conditions and Healthcare Services Use survey carried out in 2015 by the Italian National Institute of Statistics (ISTAT). To the best of our knowledge, no previous published studies have focused on the relationship between SRH and perceived economic condition in a developed country such as Italy. Moreover, as already mentioned, most previous research on subjective income has primarily focused on elderly people, while our study covers an entire population, with ages of participants ranging from 15 to more than 65 years (divided into young, working age and retired age groups). It also covers three geographical areas in Italy (North, Centre and South). This is pertinent, because although Italy has an excellent health system, there are long-standing concerns regarding health disparities between these regions [55]. For example, the availability of advanced medical equipment is lower and community care services less developed in the southern region than in the wealthier northern areas (European Portal for Action in Health Inequality).

Overall, the summarized research suggests that people’s perceptions of the adequacy of their economic resources, relative to their needs, may have important implications (in addition to objective income) for their health and well-being. Thus, elucidation of effects of perceived economic status (subjective income) on SRH is important for formulation of effective social policy and adjustment of health systems in specific areas in Italy and elsewhere.

The rest of the paper is organised as follows. Section Literature review provides a literature review. Section Empirical framework describes the empirical framework. Section Model specification presents the econometric analysis, while results are discussed in Section Results and discussion, and conclusions are presented in Section Conclusions.

## Literature review

There is very extensive literature on SRH and its determinants. The main foci have varied, but many studies have addressed relationships between SRH and health-related behaviours, often including smoking status, dietary assessments, physical activity, body mass index or obesity, and alcohol intake [2, 18, 29]. The reviewed studies provide some evidence that health behaviours affect SRH, but the relationships are ambiguous, not always in expected directions, and modulated by age, gender, and ethnicity. Further studies have used socio-demographic variables (generally gender, age, education, occupation and income) as determinants of SRH. For example, using data from the 2000 Italian National Health Interview Survey, Costa et al. [15] considered geographic variation in subjective health and presence of chronic conditions, focusing on effects of individual and area-based socioeconomic conditions across Italian regions. They found a North-South gradient in self-assessed health, mainly associated with social disadvantage (proxied by low education level). In contrast, based on a relatively small sample of household income and health data from the Bank of Italy collected in 2004, Carrieri [12] found a positive national-level relationship between individual income and SRH, but no clear socioeconomic disparity in this respect between northern and southern Italy. However, Carrieri’ study did not consider simultaneously the roles of regional and individual level characteristics.

Studies by Humphries and van Doorslaer [30] and Hernandez-Quevedo et al. [28] have also found indications of inverse links between individuals’ SRH and income, in Canada and Britain, respectively. In stark contrast, Jürges [33] found that richer respondents in Germany (with income in the 3rd or 4th quartile) tended to understate their clinical health in SRH assessments.

Besides objective income, a subjective income variable, ‘perceived income adequacy’, has been introduced as a potential determinant of SRH. However, few published studies have investigated possible effects of perceived financial hardship on health status.

Much of the existing research on subjective income has focused on elderly people who tend to report a higher degree of perceived income sufficiency than younger groups [14, 27]. Several explanations have been offered for this finding, including a general decline in expenses in late life, allowing older people to manage with lower incomes.

Cheng et al. [14] investigated the relationship between self-rated financial situation and the health status of elderly people in China and found lower rates of poor health among financially well-off respondents than among those with worse financial conditions. Similarly, Bidyadhar [5] found that perceived economic well-being was significantly associated with the SRH of people aged ≥50 years in India. Moreover, Reyes Fernández et al. [43] detected relationships between poor self-rated economic situation, poor SRH, and life dissatisfaction in Costa Rica. Accordingly, Pu et al. [42] found associations between low subjective financial satisfaction, low education and poor SRH (especially depressive symptoms) among middle-aged and elderly people surveyed in Taiwan.

As noted above, and highlighted by this brief review, studies of links between SRH and perceived economic adequacy have largely focused on older people. This is at least partly because income tends to decline in late life, prompting concern about elderly people’s perceptions of their economic resources during retirement. Therefore, more investigation of the relationship between perceived economic status and SRH is needed, particularly in Western countries and for broader age groups.

## Empirical framework

### Data source

As already mentioned, data for this study were retrieved from the national Health Conditions and Healthcare Services Use survey, carried out by the Italian National Centre of Statistics (ISTAT)^1^ in 2015. Data were collected, by questionnaire, on all individuals aged 15 years and older of approximately 16,000 households in 562 Italian municipalities, regardless of their health conditions. Information was collected through face-to-face paper and pencil interviews with each member of every family, conducted at the family home, by interviewers trained by ISTAT. The questions covered the health status, health determinants and use of the health services, together with socio-demographic context, of each individual in the interviewed families.

A two-stage sampling method was used to select municipalities. In the first stage, municipalities were stratified into large cities and small towns and villages. All the large cities were included, while small towns and villages were selected with probability proportional to their size. In the second stage, families were selected randomly from the municipal registry lists. All members of selected families were included in the sample.

### Variable definition and descriptive statistics

In this section, we define all variables used in this study and discuss the sample characteristics, which are computed at individual level and presented in Table 1.

**Table 1:** Sample characteristics (*n* = 24,149)

	Mean	S.D.
Male	0.48	0.50
Age: 1 = 15–24, 2 = 25–64, 3 = 65 years or over		
1	0.11	0.31
2	0.62	0.48
3	0.27	0.44
Income: 1 = lowest to 5 = highest quintile		
1	0.19	0.39
2	0.20	0.40
3	0.20	0.40
4	0.21	0.41
5	0.21	0.41
Educational level: 1 = Elementary school (6-11 years), 2 = Lower high school (11–14 years), 3 = High school (14–19 years), 4 = University or post-graduate (19 years-or over)		
1	0.19	0.39
2	0.31	0.46
3	0.36	0.48
4	0.14	0.34
Chronic disease	0.32	0.47
Regions: 1 = North, 2 = Central, 3 = South		
1	0.46	0.50
2	0.20	0.40
3	0.34	0.47
Perception of household economic resources: 1 = insufficient, 2 = scarce, 3 = adequate, 4 = very good	2.63	0.63
1	0.06	0.23
2	0.28	0.45
3	0.64	0.48
4	0.03	0.16
Employment status: 1 = employed, 2 = retired, 3 = unemployed, 4 = not in labour market (student, disabled, etc.)	2.18	1.24
1	0.43	0.49
2	0.22	0.41
3	0.09	0.29
4	0.26	0.44

#### Self-reported health status

We examine relationships between SRH status and socioeconomic variables, presence of chronic disease, and the influence of perceived economic resources, using an ordered probit response model. The dependent variable is self-rated health, as measured by responses to the question “How is your health in general?” on a 1–5 discrete scale: 1 = Very bad, 2 = Bad, 3 = So-so, 4 = Good and 5 = Very good. Percentages of responses (24,149 in total) in these categories are 1.8, 6.7, 22, 47.6 and 21.9%, respectively. Since the frequency of responses in extreme category 1 (Very bad) is so low, the two categories 1 and 2 are merged. This may lead to loss of information [41], but the reliability of an ordered probit model may be impaired when there are only a few entries in some categories of the ordinal variable. So, self-reported health is measured on a 1 to 4 discrete scale, coded as 1 = Very bad or bad, 2 = So-so, 3 = Good, and 4 = Very good, accounting for 8.5, 22, 47.6 and 21.9% of responses, respectively.

The independent variables include sociodemographic characteristics, chronic disease and perceived economics resources.

#### Gender

The proportion of males in the sample is 48%.

#### Age

The variable age is a categorical variable with categories of 15–17, 18–19, 20–24, 25–34, 35–44, 45–54, 55–59, 60–64, 65–74, and 75 years or over (frequencies of responses: 3.25, 2.11, 5.64, 12.44, 15.75, 18.22, 8.08, 7.66, 13.44 and 13.41%, respectively). However, it is collapsed into three categories (1 = 15–24; 2 = 25–64 and 3 = 65 years or over) to improve the association with working age classification. Age group 1 (15–24, 11%) is interpreted as ‘young’ people who may not have established themselves yet in the job market. Age group 2 (25–64, 62.2%) is interpreted as the ‘working’ group and age group 3 (65 or over, 26.8%) as the ‘retired’ group.

The classes reflect our primary interest in investigating health with a focus on people who earn different incomes and belong to different classes, such as class of education, working class and pensioners.

#### Education

Education in Italy is divided into five stages: i) Preschool from 3 to 6 years; ii) Elementary school usually from 6 years to 11; iii) Lower high school, from 11 to 14 years of age; iv) High school from 14 to 19 years of age; v) University from 19 years. The variable educational level is a categorical variable indicating respondents’ highest level of education according to 1 = Elementary school (18.5%); 2 = Lower high school (31.3%); 3 = High school (36.5%); 4 = University or post-graduate (13.6%).

#### Presence of chronic disease

The presence of chronic disease^2^ was based on self-reported chronic diseases diagnosed by the participant’s physician. Some kind of chronic disease was reported by 32.44% of the sample.

#### Region

45.56, 20.06 and 34.48% of the sample lived in the northern, central and southern regions, respectively, when interviewed.

#### Income

Participants’ income is an objective measure, coded from 1 (lowest) to 5 (highest) quintile.

In the survey, it was asked the net income for each family. Data provided by ISTAT were only expressed and coded in five quintiles.

*Perception of own economic resources*, a subjective measure of income, is measured on a discrete scale with four categories: 1 = insufficient, 2 = scarce, 3 = adequate, and 4 = very good (accounting for 5.6, 28.22, 63.5 and 2.68% of respondents, respectively).

#### Occupation

Regarding employment status, at the time of the interviews 42.89% of the respondents were employed, 21.85% retired, 9.12% unemployed and 26.14% studying, disabled or unable to work for other reasons.

## Model specification

Self-reported health status is measured on a 1–4 discrete scale. Effects of factors influencing such a subjective indicator of health status could be investigated by OLS (Ordinary Least Squares) analysis if it was a cardinal scale, but this would imply that the differences between successively increasing health status categories (e.g. 1 and 2 or 3 and 4) are all the same. This may not be true, so health status must be treated and modelled as an ordinal variable. Usually, such ratings are interpreted as choices relative to specific cut points along a continuum of a latent variable, ${y}_i^{\ast }$, which is assumed to depend on a set of independent variables:

1 $$ {y}_i^{\ast }={x}_i^{\hbox{'}}\beta +{\varepsilon}_i\kern1em \mathrm{i}=1,2\dots, N $$

where *β* is a vector of parameters, *x* is a vector of independent variables (no constant included) and index *i* denotes a specific individual. The error term ( *ε*_*i*_ ) is assumed to be independently and identically normally distributed, N (0,1).

In this study, *y* is the observed ordinal rating on a 1–4 scale or level of subjective health status. Cut points are represented as *μ*_*j*_, where *μ*_1_ < *μ*_2_ < *μ*_3_. For the general case, see for example Verbeek [50]. As already mentioned, it is assumed that the value of unobserved ${y}_i^{\ast }$ (health status on a continuous scale), relative to the cut points, defines its rating on the 1–4 discrete scale**.**

The estimated *β* coefficients are not very informative. We are usually interested in the marginal effects, for instance, effects of differences in education levels of the probability of a rating of 4, such as “good” as SRH for the ordinal response variable. Estimates could be obtained by the maximum likelihood method. However, an ordered probit model (like a logit model) is based on some restrictive assumptions. One is that coefficients of the explanatory variables are the same for all categories of the response variable (‘parallel regression’), and another is constancy of ratios of independent variables’ marginal effects on probabilities of given choices on the discrete rating scale [7, 25]. These assumptions can be tested and relaxed by applying the generalized ordered probit model [7, 25, 53, 54]. In the following, we will estimate and compare results of both the standard and generalized ordered probit model are estimated and compared in the following section.

As mentioned before, the main objective of this study is to examine the influence of perceived economic resources on subjective health status. To do so, we must control for effects of several variables. One is the ‘objective’ level of income, measured in this study in terms of quintiles of income (1 = lowest, 5 = highest). This is not an ideal measure of income, since in some cases two households or individuals with very similar income levels will fall in different adjacent quintiles. However, that is inevitable when using discrete categorical variables. The other control variables are age, sex, education level, region of residency, employment status and having/not having chronic diseases.

## Results and discussion

In a first analytical stage, we test the restrictive assumptions of the standard ordered probit model by contrasting it with the generalized version. The null hypothesis is that coefficients of the independent variables are the same for each category of the respondent variable. In our case, the null hypothesis is rejected by a likelihood-ratio test, assuming ordered probit being nested in the generalized ordered probit. Also, standard criteria of models’ performance, including Akaike’s Information Criterion (AIC), Schwarz’s Bayesian Information Criterion (BIC) and the pseudo R^2^ criterion, favour the more flexible (unrestricted) generalized ordered probit model. The final estimates of coefficients obtained from a standard ordered probit model and a generalized ordered probit model are shown in Table 2, in columns labelled OP and GOP_1 to GOP_3, respectively.

**Table 2:** Final estimation results based on ordered probit (OP) and generalized ordered probit (GOP)

Independent variables	OP	GOP_1	GOP_2	GOP_3
Male	0.148^***^ (0.016)	0.080^**^ (0.031)	0.175^***^ (0.022)	0.161^***^ (0.021)
Age group 2	− 0.886^***^ (0.030)	− 0.523^***^ (0.092)	− 0.830^***^ (0.048)	− 0.804^***^ (0.031)
Age group 3	− 1.348^***^ (0.037)	− 0.823^***^ (0.095)	−1.332^***^ (0.054)	− 1.244^***^ (0.048)
Income (quintile)	0.010 (0.006)	0.009 (0.012)	0.037^***^ (0.008)	− 0.009 (0.008)
Educational level 2	0.287^***^ (0.024)	0.268^***^ (0.025)	0.268^***^ (0.025)	0.268^***^ (0.025)
Educational level 3	0.459^***^ (0.025)	0.400^***^ (0.039)	0.496^***^ (0.029)	0.409^***^ (0.031)
Educational level 4	0.636^***^ (0.030)	0.554^***^ (0.060)	0.717^***^ (0.039)	0.571^***^ (0.038)
Chronic disease	−1.220^***^ (0.018)	−1.459^***^ (0.037)	−1.238^***^ (0.021)	− 1.013^***^ (0.031)
Region central	0.075^***^ (0.017)	0.187^***^ (0.031)	0.129^***^ (0.022)	−0.012 (0.022)
Region south	0.021 (0.021)	0.020 (0.021)	0.020 (0.021)	0.020 (0.021)
**Perc. Econ. Res. Scarce**	0.093^**^ **(0.037)**	0.201^***^ **(0.051)**	**0.038 (0.043)**	**0.039 (0.045)**
**Perc. Econ. Res. Adequate**	0.250^***^ **(0.037)**	0.402^***^ **(0.052)**	0.245^***^ **(0.042)**	0.128^***^ **(0.044)**
**Perc. Econ. Res. Very good**	0.550^***^ **(0.059)**	0.500^***^ **(0.058)**	0.500^***^ **(0.058)**	0.500^***^ **(0.058)**
Employed	0.120^***^ (0.024)	0.376^***^ (0.046)	0.184^***^ (0.029)	−0.039 (0.029)
Retired	−0.039 (0.027)	0.096^**^ (0.040)	−0.041 (0.034)	− 0.300^***^ (0.047)
Unemployed	0.113^***^ (0.033)	0.066^**^ (0.030)	0.066^**^ (0.030)	0.066^**^ (0.030)
Cut point 1	−2.510^***^ (0.050)	−2.025^***^ (0.105)	−1.084^***^ (0.064)	0.256^***^ (0.056)
Cut point 2	−1.245^***^ (0.049)			
Cut point 3	0.506^***^ (0.048)			
*N*	24,149		24,149	
LL (null)	−29,659.387		−29,659.387	
LL (model)	−23,509.355		−23,244.81	
AIC	47,056.711		46,575.61	
BIC	47,210.459		46,923.57	
Pseudo R^2^	0.21		0.22	

Perc. Econ. Res. refers to perceived economic resources; * *p* < 0.10, ** *p* < 0.05, *** *p* < 0.01

As the generalized ordered model is statistically superior for all the categorical independent variables, we examine the significance of differences between coefficients for different levels of the categorical independent variables. For the variable income, there was not found any significant difference. Partly for this reason, and partly because it is ordinal (recorded in quintiles), it is treated as an interval variable.

It should be noted that although model selection has been based, so far, purely on statistical criteria, there might be good theoretical reasons to expect coefficients of the same independent variables to differ for different outcome categories in our application. This is because the criteria and thresholds people apply when assessing their health status (the ordered dependent variable in this study) are likely to vary. For example, elderly respondents may use different frames of reference from young people when assessing their health status on a scale of, say, 1 = poor to 4 = very good. This leads to state-dependent reporting or scale of reference bias [54].

Values of the coefficients (β) of the independent variables, however, do not give precise information about effects of changes in the independent variables on the health status rating. Further calculations of the marginal effects on the probability of each rating (1–4) are needed. Based on the estimated coefficients in Table 2, we can calculate the effect of unit changes in the independent variables on the probability of different outcomes for the dependent variable.

For example for a continuous variable, say *x*_*k*_, the marginal effect on the probability of a health status rating of 4 would be, on average: $\frac{\partial P\left(y=4|x\right)}{\partial {x}_k}=\frac{\partial \left(1-\Phi \left({\mu}_3-{x}^{\hbox{'}}\beta \right)\right)}{\partial {x}_k}$ (see for example, [50]). Table 3 reports all the marginal effects.^3^

**Table 3:** Marginal effects of the independent variables on SRH outcomes 1(Very bad) – 4 (Very good)

Independent variables	1	2	3	4
Male	−0.004^*^ (0.002)	−0.050^***^ (0.006)	0.017^*^ (0.007)	0.038^***^ (0.005)
Age group 2	0.027^***^ (0.004)	0.213^***^ (0.011)	−0.033^*^ (0.013)	−0.207^***^ (0.009)
Age group 3	0.070^***^ (0.011)	0.394^***^ (0.018)	−0.248^***^ (0.018)	−0.217^***^ (0.007)
**Income (quintile)**	**−0.000 (0.001)**	−0.011^***^ **(0.002)**	0.014^***^ **(0.003)**	**−0.002 (0.002)**
Educational level 2	−0.014^***^ (0.001)	−0.068^***^ (0.006)	0.015^***^ (0.001)	0.066^***^ (0.006)
Educational level 3	−0.021^***^ (0.002)	−0.128^***^ (0.008)	0.046^***^ (0.008)	0.102^***^ (0.008)
Educational level 4	−0.022^***^ (0.002)	−0.161^***^ (0.007)	0.020 (0.012)	0.162^***^ (0.012)
Chronic disease	0.153^***^ (0.005)	0.268^***^ (0.007)	−0.223^***^ (0.008)	−0.198^***^ (0.005)
Region central	−0.010^***^ (0.002)	−0.030^***^ (0.007)	0.043^***^ (0.007)	−0.003 (0.005)
Region south	−0.001 (0.001)	−0.005 (0.005)	0.002 (0.002)	0.005 (0.005)
**Perc. Econ. Res. Scarce**	−0.010^***^ **(0.003)**	**−0.002 (0.013)**	**0.003 (0.014)**	**0.009 (0.011)**
**Perc. Econ. Res. Adequate**	−0.026^***^ **(0.004)**	−0.053^***^ **(0.013)**	0.049^***^ **(0.013)**	0.030^**^ **(0.010)**
**Perc. Econ. Res. Very good**	−0.018^***^ **(0.002)**	−0.112^***^ **(0.011)**	**− 0.015 (0.008)**	0.145^***^ **(0.020)**
Employed	−0.020^***^ (0.002)	−0.037^***^ (0.008)	0.067^***^ (0.009)	−0.009 (0.007)
Retired	−0.005^*^ (0.002)	0.018 (0.011)	0.051^***^ (0.013)	−0.064^***^ (0.009)
Unemployed	−0.004^*^ (0.002)	−0.017^*^ (0.008)	0.004^**^ (0.002)	0.016^*^ (0.008)

Perc. Econ. Res. refers to perceived economic resources: * *p* < 0.10, ** *p* < 0.05, *** *p* < 0.01

Here, however, the main interest is in effects of the two income variables on the probabilities of different SRH outcomes of health status, i.e., the objective variable (income quintile) and subjective variable (respondents’ perception of their economic situation on a discrete scale from 1 = insufficient to 4 = very good). In Table 3, we report and highlight the average marginal effects of both income variables (perceived economic resources and objective income expressed in quintile). It should be noted that statistical testing indicated that objective income had the same effect at all levels (income quintiles) on the response variable.

The results suggest that the objective income has no significant effect on the probability of a rating of SRH of 1 or 4. The probability of a rating of 2 decreases with about 1 percentage point and that of a rating of 3 increases with 1.4 percentage points. In contrast, Etilé and Milcent [22] found a positive correlation between SRH and income, strongly suggesting that poverty is negatively correlated with declared level of health.

Regarding the effect of the subjective income variable, the estimates indicate (inter alia) that perceptions of scarce, adequate and very good economic resources were respectively associated with 1, 2.6 and 1.8 percentage point declines in the probability of respondents rating their SRH in the 1 category (relative to the reference level, insufficient), when all other considered factors were equal. Conversely, perception that their economic resources were adequate was associated with an increase of 4.9 percentage points in the probability of an SRH rating of 3, all else equal. Similarly, the probability of an SRH rating of 4 increased by 14.5 percentage points when their perceived economic situation was very good, all else equal. Overall, the estimates clearly indicate that the actual income level (proxied by income quintile) did not affect the respondents’ SRH significantly, while their subjective perceptions of their economic situation had more significant effect (both statistically and practically). Our findings show that subjective income provides a measure that reflects an individual’s overall economic utility (well-being) more strongly than objective income, although individuals’ perceptions of their economic resources are clearly subjective, and very sensitive to their frames of reference. Thus, to raise the well-being/life satisfaction of individuals with insufficient perceived economic resources, policy-makers could implement measures to improve their SRH, for example by providing in-home services, and day care centres offering physical health care and psychological assistance.

The results reported in Table 3 also indicate that a number of other variables, such as age, educational level, presence/absence of chronic disease, and employment status, affect SRH more significantly than objective income. Coefficients for the effect of age on SRH show (inter alia) that probabilities of SRH ratings of 1 and 3 were respectively 2.7 and 3.3 percentage points higher and lower for respondents aged 25–64 years than for the reference (15–24 years) group, all else equal. In addition, probabilities of SRH ratings of 1, 2, 3 and 4 for ≥65-year-old respondents were 7, 39 percentage points higher and 25 and 22 percentage points lower than for the reference group, all else equal. Elderly people (≥ 65 years old) rate 1 and 2 of poor SRH than younger counterparts. Our results are in line with findings based on European, American and Italian data by van Doorslaer and Koolman [49], Kiuila and Mieszkowski [35] and Franzini and Giannoni [23], respectively and consistent with findings by Bidyadhar [5] and Cheng et al. [14] that perceived economic condition has significant effects on the SRH of elderly people.

It should be noted that Italy has the highest proportion of over-65 s in the EU and one of the most rapidly aging populations in the world [32]. Correspondingly, high (and increasing) incidence of chronic diseases could clearly have profound impacts on the health and quality of life of elderly people in Italy. We found that chronic disease increased probabilities of SRH ratings of 1 and 2 by 15 and 27 percentage points, respectively, and reduced ratings of 3 and 4 by 22 and 20 percentage points, respectively. Respondents suffering from chronic disease also reported low health levels. Thus, provision of benefits to *home caregivers* for patients with chronic disease is needed to ensure adequate social support.

 The results indicate that, all else equal, probabilities of SRH ratings of 1, 2 and 4 are respectively 1, 3 and 0.3 percentage points lower for central region, than the north region of Italy. The results also suggest that probabilities of SRH ratings of 3 and 4, are respectively 0.2 and 0.5 percentage points lower for the south region than the north region of Italy.

Educational effects included increases in probabilities of SRH ratings of 3 and 4 with increases in educational level. The coefficients of those who have university degree show that probabilities of SRH ratings 4 is higher respectively 16.2 percentage points than those who have only elementary education. This may be because highly educated individuals tend to obtain better (healthier, less stressful, more independent) employment [10, 17, 52]. They also generally earn more and can afford better housing and living standards (such as more physical activity, better diet, etc.) [31, 37, 38]. Moreover, they can count on more social relations (with doctors or informed people). Regardless of the reasons, our findings indicate that variations in education contribute more strongly to health disparities in Italy than geographical factors, in accordance with findings by Etilé and Milcent [22], Franzini and Giannoni [23] and Sarti and Rodrigez [44]. However, Costa et al. [15] found that low education has a more negative impact on SRH in southern regions and disadvantaged areas in Italy, than in northern and more privileged areas.

We also found significant differences in SRH related to working status. Employment reduced the probability of a rating of 1 by 2 percentage points and increased the probability of a rating of 3 by 6.7 percentage points (relative to the ‘other’ category), all else equal. Similarly, Böckerman and Ilmakunnas [6] found that the unemployed have lower SRH than the continually employed. Our results also show that retirement reduced the probability of a 4 rating by 6.4 percentage points, all else equal, supporting findings by Franzini and Giannoni [23] that both employees and the self-employed reported better health than those who were not working. To summarize, respondents who were working reported better health than those who were not working [19, 34, 47–49].

Respondents with university education reported better health than those with less education, but in contrast to previous findings, we detected no evidence of a strong north-south gradient in SRH, although people living in the central region of Italy had good self-rated health. This suggests that education is more important than geographical location for SRH.

The results that the unemployed had poorer SRH than those who were currently working or retired suggest that effects of factors such as education and unemployment on SRH should be more rigorously addressed by policy-makers. Awareness of the importance of education could encourage government and local institutions to promote access to education to address health disparities across different areas in Italy. Better-educated people are more likely to choose healthier lifestyles, and have more job opportunities and economic resources, accompanied by better perceived health. Additional policies could be implemented to support unemployed people (and those with inadequate perceived economic resources) with economic benefits and psychological assistance.

One important aspect is whether there are any systematic differences between the male and female groups regarding SRH and the effect of the explanatory variables. The distribution of the SRH by gender in the data indicates that males report more frequently higher rating than females. For example, 75% of males report a Good or a Very Good rating while this percentage for females is 65%. This difference is also captured by the estimated models. The model predicts that 67% (72%) of females (males) report a Good or a Very Good rating. It can be seen in Table 3 that the likelihood of reporting a Very Good (Bad) rating of SRH is 4 (5) percentage points greater (less) for an average male person than a female person, all else equal. These differences are also statistically significant.

To check whether there are also systematic differences between males and females regarding the effect of income, both objective and in terms of perceived economic resources, separate models were estimated for the two groups. The results are reported in Tables 4 and 5 in the Appendix. As for the effect of objective income there are essentially no differences. The estimated marginal effects for males (females) are − 0.000 (− 0.001), − 0.012 (− 0.011), 0.013 (0.016) and − 0.000 (− 0.004).

However, regarding the effect of perceived economic situation on SRH there can be seen some differences between males and females. For example, the results in Tables 4 and 5 indicate that, for males, perceptions of Scarce, Adequate and Very good economic resources were respectively associated with 0.8, 1.7 and 1.2 percentage point declines in the probability of respondents rating their SRH in the “Very bad” category (relative to the reference level, insufficient), all else equal. Same estimates for the females are 0.7, 2.9 and 2.4 percentage points decline. On the other extreme the differences are smaller. For males, perceptions of Scarce, Adequate and Very good economic resources were respectively associated with 0.3, 3 and 15.1 percentage point increases in the probability of respondents rating their SRH in the “Very good” category (relative to the reference level, insufficient), all else equal. Same estimates for females are 2.3, 3.4 and 13.9 percentage points. The most typical cases are when the perceived economic situation is stated as Adequate and SRH is Good by the respondent. The results indicate that, for females, it is almost 7 percentage points more likely that a person with Adequate (compared to insufficient) perceived economic situation rates her health as Good. For males it is about 2 percentage points, however statistically not significantly different from zero. This difference for the most typical cases maybe due to that while females are less likely than males to have a high rating on SRH, the effect of better perceived economic situation is greater, on the margin, to increase the likelihood of a higher SRH. One possible explanation is that male’s perceptions of their economics resources are more stable than female’s perceptions and male are more likely to perceive a stronger continuity in financial resources regardless of any changes in their objective situation compared to female [16]. Moreover, female utilize the health care services more than male [4, 11], so an increase of perceived income might affect the self-health assessment of female more than male.

## Conclusions

We investigate the effect of object and perceived economic resources, together with other socio-demographic characteristics variables on self-rated health. Our results indicate that subjective income affects SRH, at least of Italians, while objective income level (proxied by income quintiles) does not significantly affect it. Respondents who self-reported scarce economic resources to meet basic needs experienced poorer SRH than those who reported adequate and very good economic resources. Our analysis demonstrates that perceived income may strongly contribute to variations in income-related health. While self-perceived economic resources have been used to assess economic well-being and satisfaction, they can also be used to assess stress levels and related health outcomes. Our findings suggest that low subjective income adversely affects subjective health. Therefore, it is important to distinguish between effects of income and individuals’ perceptions of their economic resources or overall financial situation on their health. From a gender perspective, our results show that female are less likely to have high rating health than male. However, as female perceive an improved economic situation, on the margin, the likelihood of a higher SRH increases compared to male.

Our study confirms the intuitive links between subjective income, objective income, and health, which have often been neglected in previous research. Finally, our study shows that using self-perceived income sufficiency in addition to objective income in analyses may provide important insights that would otherwise be missed. Subjective income can provide a measure that reflects an individual’s overall economic well-being more than objective income and individuals’ perceptions of their economic resources are clearly subjective, and may also depend on many different factors which cannot identified in our analysis.

Our conclusion is that self-perceived income sufficiency can be a better indicator of the household’s SES than self-declared household income, because perception of your economic situation, although can be a relative concept, can affect more the status of your health.

It should be noted that our study has several limitations, including lack of detailed data on objective income-related variables, e.g. annual income. Furthermore, participants may have appraised their levels of resources relative to needs of their household members. Thus, more detailed and refined analysis is warranted.

## Data Availability

The datasets generated during and analysed during the current study are available in the ISTAT (Italian National Institute of Statistics) repository, http://siqual.istat.it/SIQual/visualizza.do?id=0071201

## Appendix

**Table 4:** Marginal effects of the independent variables on SRH outcomes 1(Very bad) – 4 (Very good) for males (*N* = 11,538)

Independent variables	1	2	3	4
Age group 2	0.036^***^ (0.006)	0.228^***^ (0.016)	−0.055^**^ (0.021)	−0.209^***^ (0.016)
Age group 3	0.101^***^ (0.024)	0.393^***^ (0.033)	−0.283^***^ (0.032)	−0.211^***^ (0.012)
**Income (quintile)**	**−0.000 (0.001)**	**−0.012**^*******^ **(0.003)**	**0.013**^******^ **(0.004)**	**−0.000 (0.003)**
Educational level 2	−0.010^***^ (0.002)	−0.059^***^ (0.008)	− 0.002 (0.002)	0.071^***^ (0.010)
Educational level 3	−0.017^***^ (0.002)	−0.100^***^ (0.008)	− 0.007^*^ (0.003)	0.123^***^ (0.011)
Educational level 4	−0.016^***^ (0.002)	−0.119^***^ (0.007)	− 0.055^***^ (0.009)	0.190^***^ (0.016)
Chronic disease	0.123^***^ (0.007)	0.254^***^ (0.010)	−0.159^***^ (0.011)	−0.218^***^ (0.007)
Region central	−0.010^***^ (0.002)	−0.029^**^ (0.009)	0.047^***^ (0.011)	−0.008 (0.009)
Region south	−0.003 (0.002)	−0.017 (0.010)	0.040^**^ (0.013)	−0.020^*^ (0.010)
**Perc. Econ. Res. Scarce**	**−0.008**^******^ **(0.003)**	**0.019 (0.017)**	**−0.013 (0.019)**	**0.003 (0.017)**
**Perc. Econ. Res. Adequate**	**−0.017**^*******^ **(0.005)**	**−0.038**^*****^ **(0.017)**	**0.025 (0.019)**	**0.030 (0.016)**
**Perc. Econ. Res. Very good**	**−0.012**^*******^ **(0.002)**	**−0.094**^*******^ **(0.013)**	**− 0.045**^******^ **(0.016)**	**0.151**^*******^ **(0.030)**
Employed	−0.039^***^ (0.005)	−0.097^***^ (0.016)	0.170^***^ (0.019)	−0.034^*^ (0.014)
Retired	−0.018^***^ (0.003)	−0.027 (0.018)	0.162^***^ (0.021)	−0.117^***^ (0.016)
Unemployed	−0.016^***^ (0.002)	−0.062^***^ (0.015)	0.083^***^ (0.019)	−0.005 (0.016)

Perc. Econ. Res. refers to perceived economic resources; * *p* < 0.10, ** *p* < 0.05, *** *p* < 0.01

**Table 5:** Marginal effects of the independent variables on SRH outcomes 1(Very bad) – 4 (Very good) for females (*N* = 12,611)

Independent variables	1	2	3	4
Age group 2	0.025^***^ (0.007)	0.214^***^ (0.017)	− 0.049^*^ (0.019)	−0.191^***^ (0.011)
Age group 3	0.075^***^ (0.015)	0.403^***^ (0.023)	−0.270^***^ (0.023)	−0.208^***^ (0.008)
**Income (quintile)**	**−0.001 (0.001)**	**−0.011**^******^ **(0.004)**	**0.016**^*******^ **(0.004)**	**−0.004 (0.002)**
Educational level 2	−0.017^***^ (0.002)	−0.071^***^ (0.009)	0.031^***^ (0.004)	0.058^***^ (0.008)
Educational level 3	−0.023^***^ (0.003)	−0.141^***^ (0.012)	0.083^***^ (0.011)	0.082^***^ (0.010)
Educational level 4	−0.024^***^ (0.003)	−0.185^***^ (0.012)	0.064^***^ (0.016)	0.144^***^ (0.016)
Chronic disease	0.177^***^ (0.007)	0.277^***^ (0.010)	−0.274^***^ (0.010)	−0.179^***^ (0.006)
Region central	−0.012^***^ (0.003)	−0.038^***^ (0.010)	0.056^***^ (0.011)	−0.006 (0.007)
Region south	−0.002 (0.002)	−0.009 (0.008)	0.005 (0.004)	0.007 (0.006)
**Perc. Econ. Res. Scarce**	**−0.007**^*****^ **(0.003)**	**−0.029**^*****^ **(0.013)**	**0.014**^*****^ **(0.006)**	**0.023**^*****^ **(0.010)**
**Perc. Econ. Res. Adequate**	**−0.029**^*******^ **(0.005)**	**−0.072**^*******^ **(0.015)**	**0.068**^*******^ **(0.013)**	**0.034**^******^ **(0.010)**
**Perc. Econ. Res. Very good**	**−0.024**^*******^ **(0.003)**	**−0.130**^*******^ **(0.017)**	**0.015 (0.008)**	**0.139**^*******^ **(0.026)**
Employed	−0.021^***^ (0.004)	−0.013 (0.012)	0.038^**^ (0.013)	−0.004 (0.008)
Retired	0.001 (0.003)	0.028 (0.015)	0.013 (0.017)	−0.041^***^ (0.012)
Unemployed	−0.004 (0.003)	−0.014 (0.012)	0.007 (0.005)	0.011 (0.009)

Perc. Econ. Res. refers to perceived economic resources; * *p* < 0.10, ** *p* < 0.05, *** *p* < 0.01

## Abbreviations

AIC: Akaike’s Information Criterion
BIC: Schwarz’s Bayesian Information Criterion
EC: European Commission
EHIS: European Health Interview Survey
EU: European Union
GOP: Generalized ordered probit
ISTAT: Italian National Institute of Statistics
OLS: Ordinary Least Squares
OP: Ordered probit
SES: Socioeconomic status
SRH: Self-rated health
WHO: World Health Organization

## Footnotes

1 The European Health Interview Survey (EHIS), established by European Commission Regulation (EC) 141/2013, was conducted in all Member States of the European Union to compare their situations in terms of the main aspects of the populations’ health conditions and use of health services.

2 Diseases or health problems that last at least 6 months or are expected to last at least 6 months.

3 All of the independent factors are binary variables (0/1), and the average marginal effect might be overestimated
